# Controlling Smad4 signaling with a Wip

**DOI:** 10.15252/embr.202050246

**Published:** 2020-03-18

**Authors:** Peter ten Dijke, David Baker

**Affiliations:** ^1^ Department Cell and Chemical Biology Oncode Institute Leiden University Medical Center Leiden The Netherlands

**Keywords:** Development & Differentiation, Post-translational Modifications, Proteolysis & Proteomics, Signal Transduction

## Abstract

Members of the transforming growth factor‐β (TGF‐β) family play key roles in embryogenesis and in maintaining tissue homeostasis, and their perturbation can result in a broad range of diseases. One way TGF‐β family signaling pathways are kept in check is by reversible (de)phosphorylation of intracellular Smad effectors. In this issue of *EMBO Reports*, Park *et al* [1] identify the phosphatase wild‐type p53‐induced phosphatase 1 (Wip1) as a negative regulator of TGF‐β family signaling. Mechanistically, Wip1 constrains TGF‐β family signaling through direct dephosphorylation of Thr277, an activating MAP kinase phosphorylation site located in the linker region of the common mediator Smad4.

## Graphical abstract

TGF‐β family signaling pathways are regulated by reversible phosphorylation of Smad effectors. A study in this issue identifies the phosphatase Wip1 as a novel negative regulator of TGF‐β signaling.

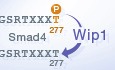

The TGF‐β family, comprising TGF‐βs, activins, nodal, and bone morphogenic proteins (BMPs), controls a broad spectrum of cellular responses via their transmembrane serine/threonine kinase type I and type II receptors. Upon ligand‐induced type I/type II receptor complex formation, intracellular receptor‐regulated (R)‐Smad effector proteins become activated following phosphorylation of two C‐terminal serine residues. These activated R‐Smads interact with Smad4, a signaling component that is shared by multiple TGF‐β family members. Smad heteromeric complexes accumulate in the nucleus where they act as sequence‐specific DNA‐binding transcription factors. Smads harbor two conserved Mad Homology (MH) domains, MH1 and MH2, located at their amino and carboxy termini, respectively. These domains are connected via a flexible proline‐rich linker region. The MH1 domain interacts with DNA, and the MH2 domain is involved in R‐Smad‐Smad4 complex formation 2.

Multiple residues of Smad4 are phosphorylated, which results in activation or inhibition of its activity. Smad4 is not directly phosphorylated by TGF‐β family receptors, rather it is subject to phosphorylation by other kinases. For example, targeting of Erk at Thr277 3 promotes Smad4 activity and glycogen synthase kinase‐3 (GSK3), which phosphorylates Thr265, Thr269, and Thr273, and inhibits Smad4 function. Whereas the details of Smad4 phosphorylation are defined, the identity of a phosphatase controlling Smad4 dephosphorylation had, until now, remained elusive. In this issue of *EMBO Reports*, Park *et al* 1 report the results of experiments designed to elucidate the effects of Wip1 on germ layer specification in early *Xenopus* embryos. Wip1 is a member of the PP2C family of serine/threonine phosphatases with an established role in a variety of processes including aging, neurogenesis, immunity, and tumorigenesis 4. The authors found that during embryogenesis, Wip1 functions to restrict mesoderm formation, nodal/activin (TGF‐β like ligands)‐induced expression of dorsal and ventral mesodermal markers, as well as BMP‐induced ventral target gene expression 1. Moreover, ectopic expression of Wip1 stimulated neural differentiation (indicated by decreased BMP signaling), whereas Wip1 depletion promoted epidermal differentiation (consistent with increased BMP signaling). Unlike wild‐type Wip1, a catalytically inactive Wip1 mutant was unable to rescue the Wip1 depletion phenotypes, suggesting that its catalytic activity is required for its normal function in embryogenesis. Consistently, Wip1 inhibited, in a phosphatase activity‐dependent manner, TGF‐β‐induced responses in human cells (Fig 1).

**Figure 1:**
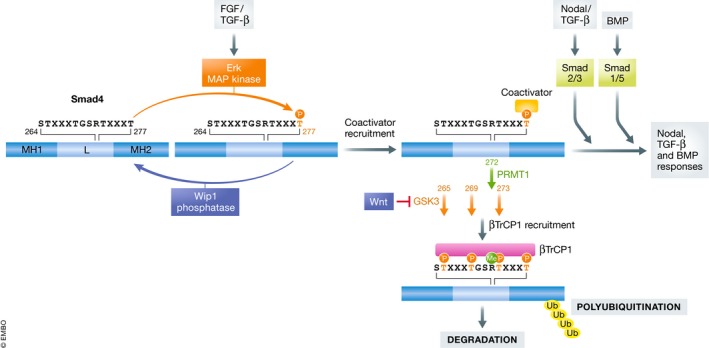
Wip1 dephosphorylates Smad4 at Thr277 in the linker region, thereby inhibiting TGF‐β family signaling responses Wip1 dephosphorylates Thr277 in the linker region of Smad4, which is phosphorylated by the MAP kinase Erk in response to FGF and TGF‐β. Thr277 phosphorylation facilitates the interaction with coactivators and thereby promotes TGF‐β and BMP signaling. Moreover, Thr277 phosphorylation primes sequential phosphorylation of Thr273, Thr269, and Thr265 by GSK3. PRMT1‐induced Arg272 methylation is required for Smad4 phosphorylation by GSK3. These phosphorylation events generate a phosphodegron recognized by βTrCP. By dephosphorylating Smad4 Thr277, WIP1 not only inhibits Smad4 transcriptional function, but also prevents Smad4 degradation. MH, Mad Homology domain; L, linker.

Since Wip1 antagonizes both TGF‐β and BMP signaling, Park *et al* 1 reasoned that the common mediator Smad4 could be a Wip1 substrate. They show that (nuclear) Wip1 interacted with Smad4 in a ligand‐dependent manner and inhibited the interaction between R‐Smads and Smad4 leading to Smad4 export. Importantly, the authors showed that Wip1 directly dephosphorylated Thr277 located in the linker region of Smad4 (Fig 1). In support of this, they found that expression of a Smad4 mutant, harboring a Glu277 phospho‐mimic residue, but not an Ala277 residue that abolishes phosphorylation, rescued the effects of ectopic expression of Wip1. These data support the view that Wip1 inhibits the activity of Smad4 by dephosphorylating Thr277. Consistently, Thr277 was previously shown to be phosphorylated by Erk in response to FGF and TGF‐β, resulting in recruitment of coactivators (such as P300 and CBP) and nuclear enrichment of Smad4 3, 5. In this model, Thr277 phosphorylation‐dependent activation of TGF‐β family signaling would be counterbalanced by Wip1‐dependent Thr277 dephosphorylation. Such a mechanism could fine‐tune TGF‐β family signaling (Fig 1).

Other studies have shown that Thr277 phosphorylation ultimately results in proteasomal degradation of Smad4: Thr277 phosphorylation primes Smad4 for sequential phosphorylation of Thr273, Thr269, and Thr265 by GSK3, thereby creating a recruitment site for the βTrCP1 E3 ubiquitin ligase. In addition, methylation at Arg272 by protein arginine methyltransferase 1 was shown to be required for GSK3‐mediated phosphorylation of Smad4 5, 6 (Fig 1). In line with these results, Park *et al* 1 now found that Wip1 antagonized FGF‐induced Erk kinase Smad4 Thr277 phosphorylation and prevented Smad4 degradation. The latter effect was also observed following Wnt stimulation, which inhibits GSK3‐induced phosphorylation and prevents recruitment of the βTrCP1 (Fig 1). Thus, by protecting Smad4 from degradation (similarly to Wnt stimulation), Wip1 could preserve a pool of cytoplasmic Smad4 that is primed for promoting signaling. Of note, Smad4 is also monoubiquitinated, which inhibits its ability to interact with R‐Smads, and promotes its nuclear export. Thr277 phosphorylation was found to enhance the interaction with the deubiquitinating enzyme USP9x, which reverses monoubiquitination and thereby enables nuclear retention of Smad4 and active TGF‐β/Smad signaling 7. By dephosphorylating Thr277, Wip1, which is mainly found in the nucleus, may oppose the effect of USP9x on Smad4 function.

Besides Smad4, a large number of other Wip1 substrates were previously identified including p53 Ser15, ataxia telangiectasia mutated (ATM) kinase Ser1981, and p38 MAP kinase Thr182 4. p53 can act as a Smad partner in certain TGF‐β/activin‐induced transcriptional responses 8. However, Park *et al* 1 found no effect on p53‐induced expression of mesodermal markers in *Xenopus* embryos upon mis‐expression of Wip1, suggesting that the negative regulation of TGF‐β signaling by Wip1 is not mediated by blocking p53‐associated Smad co‐activator functions. The activity of ATM is negatively regulated by Wip1. ATM activation is strongly induced after DNA damage, which has been shown to stabilize the TGF‐β type II receptor 9. It will be interesting, therefore, to explore whether Wip1 participates in a negative feedback loop to regulate TGF‐β signaling after DNA damage. In certain cells, p38 MAP kinase can be activated downstream of TGF‐β family receptors 2. Wip1 may thus not only inhibit canonical Smad signaling, but also the non‐canonical p38MAK kinase signaling pathway, to blunt TGF‐β family‐induced responses.

Wip1 expression is upregulated by stress signals and NFκB signaling, and its function is regulated by post‐translational modifications as well as by interacting proteins 4. This study raises the question as to whether certain extrinsic or intrinsic cellular cues that affect Wip1 activity exert part of their cellular effects by inhibiting TGF‐β family signaling responses. In this light, it will be of interest to examine whether Wip1 protein expression, activity, and/or localization are regulated by TGF‐β family members as part of a feedback response.

The *PPM1D* gene encoding Wip1 is amplified, mutated, and/or highly expressed in multiple cancer subtypes. *PPM1D* has been classified as being an oncogene as it inactivates many tumor suppressor proteins and stimulates cell proliferation 4. Smad4 acts as a tumor suppressor in pancreatic and colon cancer, and the inhibitory effect of Wip1 on Smad4 function appears to add Smad4 to the list of Wip1 tumor suppressor substrates. However, like TGF‐β, Smad4 has a dual role in breast and other cancer subtypes 10. Depending on cancer stage, Smad4 can mediate cytostasis (early stage) or promote invasion and metastasis (late stage). In agreement with this notion, Park and colleagues found that depletion of Wip1 promoted TGF‐β‐induced growth arrest of human embryonic kidney cells and also enhanced TGF‐β‐induced migration of aggressive human breast cancer cells 1. Small molecule Wip1 phosphatase inhibitors are currently being explored for cancer therapy. Given the findings presented by Park *et al*, judicious cancer patient selection, based upon Smad4 status and tumor type, could help to ensure that the pharmacological targeting of Wip1 will only potentiate the tumor suppressor function of Smad4.
